# Exposure to per- and poly-fluoroalkyl substances and associations with embryo quality and adverse pregnancy outcomes: a prospective cohort study

**DOI:** 10.3389/fpubh.2026.1783940

**Published:** 2026-03-06

**Authors:** Jiahui Wang, Zhe Li, Kuona Hu, Jingmei Hu, Ting Jiang, Jia Liao, Qian Zhang, Lijing Sun, Linlin Cui, Rong Chen, Tianxiang Ni, Wei Zhou

**Affiliations:** 1 Department of Gynecology and Obstetrics, Peking Union Medical College Hospital, Chinese Academy of Medical Sciences & Peking Union Medical College, National Clinical Research Center for Obstetric & Gynecologic Diseases, Beijing, China; 2 State Key Laboratory of Reproductive Medicine and Offspring Health, Center for Reproductive Medicine, Institute of Women, Children and Reproductive Health, Shandong University, Jinan, Shandong, China; 3 National Research Center for Assisted Reproductive Technology and Reproductive Genetics, Shandong University, Jinan, Shandong, China; 4 Key Laboratory of Reproductive Endocrinology (Shandong University), Ministry of Education, Jinan, Shandong, China; 5 Shandong Technology Innovation Center for Reproductive Health, Jinan, Shandong, China; 6 Shandong Provincial Clinical Research Center for Reproductive Health, Jinan, Shandong, China; 7 Shandong Key Laboratory of Reproductive Research and Birth Defect Prevention, Jinan, Shandong, China; 8 Research Unit of Gametogenesis and Health of ART-Offspring, Chinese Academy of Medical Sciences (No. 2021RU001), Jinan, Shandong, China; 9 Department of Urology, Shandong Provincial Hospital, Shandong University, Jinan, Shandong, China; 10 Department of Ultrasound, Qilu Hospital of Shandong University, Jinan, Shandong, China

**Keywords:** embryo, intracytoplasmic sperm injection (ICSI), *in vitro* fertilization (IVF), per- and poly-fluoroalkyl substances (PFAS), pregnancy

## Abstract

**Background:**

Per- and poly-fluoroalkyl substances (PFASs) are a group of synthetized industrial pollutants which have been detected worldwide in both environment and humans and gradually raised public health concerns. Previous studies suggested that PFASs had reproductive toxicity and may do harm to pregnancy and children. However, it is still uncertain that whether effect of PFAS exposure on offsprings begins from embryo quality or post-implantation pregnancy outcomes. This study explores the association between exposure to PFASs and embryo quality or pregnancy outcomes to promote maternal–infant health.

**Methods:**

This study included 246 women who underwent their first *in vitro* fertilization (IVF) or intracytoplasmic sperm injection (ICSI) cycles from July 2017 to August 2018 and measured concentrations of 23 PFAS congeners of follicular fluid samples. Generalized linear regression model with ln-transformed concentration values was performed to evaluate the association between individual PFAS congener and embryo quality and pregnancy outcomes. Weight quantile sum (WQS) regression and Bayesian kernel machine regression (BKMR) were used to estimate mixed effect of PFAS exposure on the above outcomes.

**Results:**

PFAS exposure had no significant association with high-quality blastocyst rate. Whereas, PFAS exposure had significantly adverse effect on miscarriage and live birth rate with the predominant risk of perfluoro-n-butanoic acid (PFBA) (For miscarriage, Q4 vs. Q1 OR: 6.44 (95%CI: 1.47, 33.72), Adjusted *p* = 0.03; For live birth, Q4 vs. Q1 OR: 0.35 (95%CI: 0.14, 0.82), Adjusted *p* = 0.04). BKMR model also suggested that there was potential joint effect between mixed PFAS chemicals and miscarriage or live birth.

**Conclusion:**

For women undergoing IVF/ICSI, exposure to PFASs was associated with higher miscarriage and lower live birth rate but had no adverse effect on embryo quality.

## 1 Introduction

Per- and Poly-fluoroalkyl substances (PFAS) are a class of artificial synthetic compounds possessing great thermal and chemical stability as well as exceptional amphiphilicity (1) thus have been worldwide applied in non-stick coating materials, textile, food packaging (2), consumer products (3), etc. However, these unique physicochemical properties also make PFAS easily mobile within soil and water (4, 5), even the air (6) obviously generating long-term environmental contamination. Humans are susceptible to ambient PFAS through the daily interactions between external environment and human bodies (7). Therefore, considering the long half-lives (8, 9) and biological accumulation (10), concerns of PFAS on reproductive health gradually emerge (11).

Infertility is the primary issue of reproductive health as it occurs in approximately 10–15% of couples at reproductive age all around the world (12). It is defined as unsuccessful pregnancy of couples after at least one-year sexual intercourse without contraception (13). Although assisted reproductive technology (ART) finds a solution for a few affected couples, there are still 15% of infertile couples have “unexplained infertility” and environmental factor is suggested as an explanation (14, 15). In addition, PFAS exposure was noted to increase the risk of infertility (14, 16) and have the association with adverse pregnancy outcomes, such as poor embryo quality (17–19), pregnancy losses (20, 21) and preterm birth (20, 22), etc. However, the above conclusions remain controversial (7, 16, 23, 24). Part of the issue is that PFAS concentrations were mostly detected from serum samples which were collected on varying days (7) and may have a large fluctuation. Therefore, convicing evidence is needed to better demonstrate the critical windows for maternal–infant health under PFAS exposure.

Follicular fluid has been verified the detection of PFAS (16, 19, 25, 26) and these samples were collected at a relatively standardized time point, the day of oocyte retrieval during ART procedure (7, 16, 19). Follicular fluid baths oocytes and better reflects oocyte microenvironment thus is suitable to elaborate the relationship between PFAS and embryo quality as well as pregnancy outcomes. Previous studies with follicular fluid samples have provided some insights. For example, Hong et al. (7) found a relatively high tendency of PFAS through blood-follicular barrier but showed no associations between PFAS concentrations and target ART outcomes (e.g., embryo quality and early pregnancy outcomes). The currently largest sample size study of embryos conducted by Zeng et al. (19) involved 8 PFAS and linked them with poorer embryo quality. Notably, existing studies have several limitations, including the narrow spectrum of detectable PFAS and insufficient data on embryo quality and pregnancy outcomes (7, 16, 19, 25). A particular issue is the lack of embryo development data beyond cleavage-stage embryo, which refers to day 3 embryo following fertilization (7, 16, 19). Day 5 embryos, known as “blastocysts,” possess better embryo vailibility and improved live birth rate compared to cleavage-stage embryos (27) thus are gradually transferred as a trend in ART treatment.

Therefore, this prospective cohort study included women receiving fresh embryo transfer cycles of ART and aimed to investigate the impact of exposure to PFAS including legal and emerging PFASs on both embryo quality and pregnancy outcomes. The findings would give much more reliable evidence to infertility etiology and develop guidance on maternal and infant health.

## 2 Materials and methods

### 2.1 Participants selection

A total of 246 female participants were recruited from Hospital for Reproductive Medicine Affiliated to Shandong University for their first fresh embryo transfer cycles of *in vitro* fertilization (IVF) or intracytoplasmic sperm injection (ICSI) from July 2017 to August 2018 (Supplementary Figure S1). The study was approved by Ethics Committee of Center for Reproductive Medicine of Shandong University (Ethical Review No. 74, 2023) and all participants have signed informed consents. The inclusion criteria were as followed: (1) aged 20–48 years old; (2) intention to undergo their first IVF or ICSI fresh embryo transfer cycles. The exclusion criteria included the following: (1) woman with uterine abnormalities or hydrosalpinx; (2) women with endocrine and metabolism disorders; (3) with pregnancy-contraindicated drug uses; (4) recipients of donor eggs.

### 2.2 Assisted reproductive technology (ART) strategies and samples collection

Sociodemographic and clinical data was collected at the beginning of ART cycles. Baseline serum hormone levels were measured by chemiluminescence immunoassay (Roche Diagnostics, Basel, Switzerland) at days 1–3 of the menstrual cycle while peak estradiol levels (E_2_) were measured by the same method at the second day after oocyte retrieval. Controlled ovarian stimulation protocols were selected individually by experienced clinicians. The specific details of long agonist protocols, short agonist protocols and antagonist protocols were described previously (28). Other protocols represent mild stimulation protocol and progestin-primed ovarian stimulation protocol. In brief, mild stimulation protocol starts with human menopausal gonadotropin (hMG) or letrozole to induce ovulation until the diameter of more than two follicles exceeds 18 mm. Progestin-primed ovarian stimulation protocol starts pituitary down-regulation at day 3 of the menstrual cycle with daily progesterone administration until the diameter of more than two follicles exceeds 18 mm. For each protocol above, oocytes were retrieved by transvaginal ultrasonography at the time of 34–36 h after trigger and follicular fluid samples were collected from the first leading dominant follicle by aspiration. Bloody fluid or diluted fluid was excluded to avoid contamination. Qualified samples were centrifugated subsequently and their supernatant was stored at −80 °C.

### 2.3 PFAS measurements

Follicular fluid samples were examined in Ministry of education-Shanghai key laboratory of children’s environmental health at Xinhua Hospital affiliated to Shanghai Jiao Tong University according to previously reported methods (29). A total of 23 PFAS congeners were measured including 6:2 chlorinated polyfluorinated ether sulfonic acid (CLP62), 8:2 chlorinated polyfluorinated ether sulfonic acid (CLP82), hexafluoropropylene oxide dimer acid (HFPODA), perfluoro-n-butanoic acid (PFBA), potassium perfluoro-1-butanesulfonate(PFBS), perfluoro-n-decanoic acid (PFDA), perfluorododecanoic acid (PFDoA), perfluoro-n-heptanoic acid (PFHpA), sodium perfluoro-1-heptanesulfonate (PFHpS), perfluoro-n-hexanoic acid (PFHxA), linear perfluorohexanesulfonate (nPFHxS), branched perfluorohexanesulfonate (BrPFHxS), perfluoro-n-nonanoic acid (PFNA), perfluoro-n-octanoic acid (PFOA), linear sodium perfluoro-1-octanesulfonate (nPFOS), potassium perfluoro-1-methylheptanesulfonate (PFOS1m), potassium perfluoro-3-methylheptanesulfonate (PFOS3m), potassium perfluoro-4-methylheptanesulfonate (PFOS4m), potassium perfluoro-5-methylheptanesulfonate (PFOS5m), potassium perfluoro-6-methylheptanesulfonate (PFOS6m), sodium perfluoro-1-pentanesulfonate (PFPeS), perfluoro-n-pentanoic acid (PFPeA), perfluoro-n-undecanoic acid (PFUnDA) (Supplementary Table S1). As PFOS3m, PFOS4m and PFOS5m coeluted together on chromatogram, their concentrations were added together and abbreviated as Mpfos345. 3 chemicals whose detection rate less than 85% were excluded from further analysis (i.e., PFDoA, BrPFHxS and PFPeA). Besides, the concentrations of PFAS congeners below the limit of detection (LOD) were replaced by the value of LOD/ √2 (Supplementary Table S2).

### 2.4 Outcome measurements

Fertilization methods included IVF and ICSI. Briefly, IVF involves *in vitro* co-incubation of sperm and a single oocyte overnight. ICSI involves the direct microinjection of a single sperm into a mature oocyte under controlled laboratory conditions. After fertilization by IVF or ICSI, all embryos were observed and scored by experienced embryologists based on Brinsden (30) or Gardner (31) Criteria who were also blinded to the PFAS measurements. MII oocyte rate, oocyte fertilization rate, high-quality cleavage embryo rate and high-quality blastocyst rate were recorded and high-quality blastocyst rate was calculated as the primary embryo quality outcomes. Biochemical pregnancy, clinical pregnancy, miscarriage and live birth were recorded. Thereinto, miscarriage and live birth reflected the influence from maternal factors and embryo viability so were regarded as the primary pregnancy outcomes and performed further sub-analysis, respectively. In detail, oocyte fertilization rate was defined as the number of two-pronuclear (2PN) zygotes divided by the number of total retrieved oocytes. High-quality cleavage embryo rate was defined as the amount of Day 3 embryos better than grade 3 with 7–10 cells divided as the number of 2PN zygotes. High-quality blastocyst rate was defined as the number of blastocysts equal to or better than 4 BC divided by the number of 2PN zygotes. Biochemical pregnancy was defined as serum *β*-human chorionic gonadotropin level >10 IU/L at 14 days after embryo transfer. Clinical pregnancy was defined as existence of gestational sac in utero at 7 weeks’ gestation. Miscarriage was defined as clinical pregnancy loss before 28 weeks’ gestation. Live birth was defined as successful pregnancy after 28 weeks’ gestation.

Pregnancy complications and neonatal outcomes were presented as additional descriptions of study population in our study. Here, pregnancy complications included gestational diabetes and pregnancy-induced hypertension while neonatal outcomes included gestational weeks at birth, preterm birth, infant sex, birth weight and low birth weight. The above information was all self-reported and detailed definition was described in Supplementary Table S3.

### 2.5 Quality control

Quality control procedures were conducted to ensure the data credibility. Briefly, the standard PFAS and isotope internal standards were purchased from Wellingtong Laboratories (Guelph, Ontario, Canada) with purity over 98%. For every 20 samples, one blank and two quality control samples of high and low concentrations (1 ppb and 10 ppb) were added to monitor analytical performance. The recovery rates of the target PFAS ranged from 70 to 130%. The precision and variation were 20 and 15%, respectively. The relative standard deviation for each PFAS congener were all within 20%.

### 2.6 Statistical analyses

Continuous variables were described by median (interquartile range) while categorial variables were described by number (percentage). To reduce the influence of outliers and normalize the skewed distributions, concentrations of PFAS congeners were ln-transformed at the beginning (32, 33). Pearson’s correlation test was performed to estimate the correlations between each PFAS congener. Generalized linear regression model was used to estimate the association between individual PFAS congener and embryo quality or pregnancy outcomes. The primary embryo outcome was high-blastocyst rate which was analyzed as a continuous variable using linear regression while primary pregnancy outcomes included miscarriage and live birth which were, respectively, treated as categorical variables for subsequent logistic regression analysis. Weight quantile sum (WQS) regression and Bayesian kernel machine regression (BKMR) were performed to evaluate mixed effect of PFAS exposure on the above outcomes. All BKMR models were run at 10,000 iterations to ensure model robustness. Based on previous literature (19, 34), some factors were considered as confounders then adjusted in the further analysis which included women’s age at oocyte retrieval, paternal age, infertility duration, body mass index (BMI), infertility diagnosis, parity and stimulation protocol. Directed acyclic graph was also depicted associations among the aforementioned indicators and confounders (Supplementary Figure S2). Multiple hypothesis testing correction was conducted using the false discovery rate. *p* value less than 0.05 was considered statistically significant. All statistical analyses were performed using R (R Foundation for Statistical Computing, Version 4.2.3, Vienna, Austria).

## 3 Results

### 3.1 Baseline characteristics of study population

Sociodemographic and clinical characteristics of 246 participants were displayed in Table 1. As shown, the average age of participants was 31.8 and interquartile range was from 29.0 to 38.1 years old. The average BMI was 24.9 kg/m^2^ with interquartile range from 21.7 to 27.6 kg/m^2^. The mean infertility duration was 3.0 years with interquartile range from 2.4 to 4.9 years. Besides, 63.4% of participants were nulliparous. The average levels of basic hormone were all in normal range. In terms of the etiology of infertility, ovulatory dysfunction, impaired gamete transport and advanced age were summarized as female factors while oligo-, atheno- and terato-spermia were summarized as male factors. Here, female factors were observed in 73.2% of participants, male factors were observed in 12.8% while the others included couples with biparental factors, chromosomal diseases or unexplained infertility. Fertilization method in this study only included IVF and ICSI, which were applied in 75.6% of participants and 24.4% of participants, respectively.

**Table 1:** Sociodemographic and clinical factors among study population (*n* = 246).

Characteristics	Values
Sociodemographic factors	
Age (y)^a^	31.8 (29.0, 38.1)
BMI (kg/m^2^)^a^	24.9 (21.7, 27.6)
Infertility duration (y)^a^	3.0 (2.0, 4.9)
Education levels^b^	
Junior school graduate or below	89 (36.2)
High school graduate/GED or equivalent	105 (42.7)
College graduate or above	52 (21.1)
Parity^b^	
Nulliparous	156 (63.4)
Parous	90 (36.6)
Clinical factors	
Basic hormone	
FSH (IU/L)^a^	6.5 (5.5, 7.9)
LH (IU/L)^a^	4.5 (3.3, 6.1)
Estradiol (pg/mL)^a^	35.9 (27.9, 44.5)
PRL (ng/mL)^a^	15.0 (11.0, 20.5)
Total testosterone (ng/dL)^a^	22.5 (14.3, 32.8)
Initial infertility diagnosis^b^	
Only male infertility factor	31 (12.6)
Only female infertility factor	180 (73.2)
Others	35 (14.2)
Ovarian stimulation protocol^b^	
Long GnRH agonist	134 (54.5)
GnRH antagonist protocol	29 (11.8)
Short GnRH protocol	81 (32.9)
Other protocol	2 (0.8)
Fertilization method^b^	
*In vitro* fertilization	186 (75.6)
Intracytoplasmic sperm microinjection	60 (24.4)
Endometrium Thickness at the day of hCG (cm)^a^	1.0 (1.0, 1.2)
Peak estradiol level (pg/mL)^a^	2261.5 (1741.5, 2897.2)
Total oocytes retrieved^a^	9.0 (6.0, 12.0)
MII oocytes retrieved^a^	7.0 (5.0, 11.0)
MII oocyte rate (%)^a^	94.1 (83.3, 100.0)
Fertilization outcome	
Oocyte fertilization rate (%)^a^	66.7 (50.0, 77.8)
The number of high-quality cleavage embryos^a^	3.0 (1.0, 4.0)
High-quality cleavage embryos rate (%)^a^	50.0 (33.0, 80.0)
The number of high-quality blastocysts^a^	1.0 (0.0, 3.0)
High-quality blastocysts rate (%)^a^	25.0 (0.0, 50.0)
Pregnancy outcome	
Biochemical pregnancy^c^	126/208 (60.6)
Clinical pregnancy^c^	113/208 (54.3)
Miscarriage^c^	27/113 (23.9)
Live birth^c^	86/208 (41.3)

BMI, body mass index; GED, general educational development; FSH, follicle stimulating hormone; LH, luteinizing hormone; PRL, prolactin; GnRH, gonadotropin-releasing hormone; hCG, human chorionic gonadotropin; MII, metaphase II.

a Median (interquartile range).

b *n* (%).

c *n*/total (%).

On average, 9 oocytes were retrieved per capita per cycle. 25.0% of oocytes were able to develop into high-quality blastocysts. Among 208 women with transferrable embryos, 54.3% achieved clinical pregnancy and 23.9% of that figure ended in miscarriage; 41.3% of these 208 participants achieved live birth.

### 3.2 PFAS concentrations in follicular fluid

The median and interquartile concentrations of 18 PFAS congeners whose detection rate more than 85% in follicular fluid were summarized in Supplementary Table S2. Among them, PFOA had the highest mean concentration (4.891 ng/mL), followed by nPFOS (1.928 ng/mL) and its alternative CLP62 (1.136 ng/mL). Pearson correlation matrix for ln-PFAS concentrations in Supplementary Figure S3 reflected the correlation between each PFAS congener and the coefficients were ranged from −0.14 to 0.93. Most congeners had strong correlation with each other except CLP82, HFPODA, PFBA, PFBS and PFHpA (r < 0.50).

### 3.3 Associations between single PFASs and blastocyst quality/pregnancy outcomes

There were no associations between single PFAS and high-quality blastocyst rate after adjusting potential confounders in generalized linear regression model (Table 2).

**Table 2:** Estimated differences of single exposure in high-quality blastocyst rate (percentage) at quantiles of ln-PFAS concentrations (nanograms per milliliter) measured in follicular fluid by generalized linear regression model (*n* = 246).

PFAS	*β* (95%CI)				P for trend	Adjusted P^a^
	Q1	Q2	Q3	Q4		
CLP62	Reference	0.11 (0.02, 0.21)	−0.04 (−0.13, 0.06)	−0.03 (−0.13, 0.06)	0.12	0.28
CLP82	Reference	0.04 (−0.06, 0.14)	−0.05 (−0.15, 0.05)	−0.04 (−0.13, 0.06)	0.17	0.26
HFPODA	Reference	−0.11 (−0.20, −0.01)	−0.11 (−0.21, −0.02)	−0.03 (−0.12, 0.07)	0.56	0.81
PFBA	Reference	0.06 (−0.03, 0.16)	0.11 (0.02, 0.21)	0.00 (−0.09, 0.10)	0.67	0.81
PFBS	Reference	−0.03 (−0.12, 0.07)	0.00 (−0.10, 0.09)	−0.05 (−0.14, 0.05)	0.44	0.49
PFDA	Reference	0.03 (−0.06, 0.12)	0.00 (−0.10, 0.09)	−0.06 (−0.16, 0.03)	0.14	0.33
PFHpA	Reference	0.04 (−0.05, 0.14)	−0.07 (−0.17, 0.03)	−0.07 (−0.16, 0.03)	0.04	0.11
PFHpS	Reference	0.01 (−0.09, 0.10)	0.01 (−0.09, 0.11)	0.01 (−0.09, 0.10)	0.91	0.88
PFHxA	Reference	−0.02 (−0.11, 0.08)	−0.05 (−0.14, 0.05)	−0.05 (−0.15, 0.04)	0.21	0.25
nPFHxS	Reference	−0.07 (−0.16, 0.03)	0.02 (−0.07, 0.12)	0.01 (−0.09, 0.11)	0.45	0.59
PFNA	Reference	0.01 (−0.09, 0.10)	0.00 (−0.10, 0.09)	−0.05 (−0.14, 0.05)	0.30	0.53
PFOA	Reference	0.06 (−0.03, 0.16)	0.07 (−0.03, 0.16)	−0.02 (−0.12, 0.07)	0.67	0.86
nPFOS	Reference	0.06 (−0.03, 0.16)	0.06 (−0.03, 0.15)	−0.06 (−0.15, 0.04)	0.28	0.52
PFOS1m	Reference	−0.02 (−0.11, 0.08)	0.03 (−0.06, 0.13)	0.00 (−0.10, 0.10)	0.77	0.82
Mpfos345	Reference	0.05 (−0.04, 0.14)	0.09 (−0.01, 0.18)	0.01 (−0.09, 0.10)	0.68	0.73
PFOS6m	Reference	0.06 (−0.04, 0.15)	0.08 (−0.02, 0.17)	0.03 (−0.06, 0.13)	0.42	0.51
PFPeS	Reference	−0.01 (−0.10, 0.08)	0.06 (−0.03, 0.16)	−0.07 (−0.16, 0.03)	0.42	0.63
PFUnDA	Reference	−0.01 (−0.11, 0.09)	−0.03 (−0.12, 0.06)	−0.04 (−0.13, 0.06)	0.41	0.72

PFAS, per- and poly-fluoroalkyl substances; *β*, regression coefficient; CI, confidence interval; BMI, body mass index. Models adjusted for women’s age at oocyte retrieval, paternal age, infertility duration, BMI, infertility diagnosis, parity, stimulation protocol.

a Adjusted with multiple hypothesis testing correction.

To further explore embryo viability and the influence of maternal factors, 208 participants were included in the analysis for live birth after excluding 38 participants whose transfer cycles were cancelled due to the lack of transferable embryos. Additionally, we included 113 participants who achieved clinical pregnancy in the analysis for miscarriage (Supplementary Figure S1). Significant positive associations were observed between PFBA and PFHpA exposure and miscarriage in the highest percentile concentrations (PFBA: Q4 vs. Q1 OR:6.44 (95%CI: 1.47, 33.72), adjusted *p* = 0.03; PFHpA: Q4 vs. Q1 OR: 5.67 (95%CI: 1.39, 29.83), adjusted *p* = 0.04) (Table 3). Table 4 showed negative association between PFBA and live birth in the highest percentile concentrations (Q4 vs. Q1 OR: 0.35 (95%CI: 0.14, 0.82), adjusted p = 0.04).

**Table 3:** Estimated differences of single exposure in miscarriage at quantiles of ln-PFAS concentrations (ng/mL) measured in follicular fluid by generalized linear regression model (*n* = 113).

PFAS	OR (95%CI)				P for trend	Adjusted P^a^
	Q1	Q2	Q3	Q4		
CLP62	Reference	0.79 (0.20, 3.23)	0.65 (0.15, 2.80)	1.44 (0.38, 5.72)	0.63	0.81
CLP82	Reference	0.81 (0.17, 3.68)	0.87 (0.20, 3.76)	1.93 (0.51, 7.81)	0.31	0.39
HFPODA	Reference	1.60 (0.39, 6.93)	2.03 (0.54, 8.23)	1.10 (0.26, 4.78)	0.81	0.90
**PFBA**	Reference	2.40 (0.54, 11.87)	3.99 (0.99, 18.89)	**6.44 (1.47, 33.72)**	**0.01**	**0.03**
PFBS	Reference	0.36 (0.08, 1.45)	0.62 (0.16, 2.35)	1.15 (0.28, 4.65)	0.70	0.89
PFDA	Reference	0.45 (0.11, 1.79)	0.82 (0.19, 3.42)	1.10 (0.28, 4.36)	0.65	0.83
**PFHpA**	Reference	1.21 (0.23, 7.03)	1.56 (0.33, 8.72)	**5.67 (1.39, 29.83)**	**0.01**	**0.04**
PFHpS	Reference	0.27 (0.06, 1.09)	1.52 (0.42, 5.62)	0.36 (0.07, 1.58)	0.64	0.83
PFHxA	Reference	1.09 (0.26, 4.73)	2.83 (0.77, 11.24)	0.71 (0.15, 3.27)	0.88	0.90
nPFHxS	Reference	0.53 (0.13, 2.02)	0.63 (0.17, 2.29)	0.52 (0.12, 2.12)	0.42	0.53
PFNA	Reference	1.01 (0.26, 3.84)	1.03 (0.28, 3.85)	0.65 (0.14, 2.71)	0.61	0.78
PFOA	Reference	0.55 (0.14, 2.05)	0.38 (0.09, 1.44)	0.55 (0.14, 2.06)	0.32	0.42
nPFOS	Reference	0.64 (0.17, 2.36)	0.19 (0.02, 1.01)	1.67 (0.46, 6.23)	0.66	0.85
PFOS1m	Reference	0.18 (0.04, 0.76)	0.54 (0.15, 1.83)	0.44 (0.10, 1.79)	0.39	0.50
Mpfos345	Reference	0.46 (0.11, 1.87)	1.24 (0.33, 4.80)	0.83 (0.20, 3.39)	0.83	0.95
PFOS6m	Reference	0.30 (0.06, 1.23)	0.76 (0.20, 2.85)	0.98 (0.25, 3.87)	0.74	0.95
PFPeS	Reference	2.33 (0.54, 11.87)	0.78 (0.16, 3.97)	2.13 (0.48, 11.02)	0.74	0.95
PFUnDA	Reference	0.36 (0.08, 1.40)	0.63 (0.14, 2.52)	0.69 (0.19, 2.46)	0.78	0.89

PFAS, per- and poly-fluoroalkyl substances; OR, odds ratio; CI, confidence interval; BMI, body mass index. Models adjusted for women’s age at oocyte retrieval, paternal age, infertility duration, BMI, infertility diagnosis, parity, stimulation protocol.

a Adjusted with multiple hypothesis testing correction.

Bold values indicate statistical significance at the *p* < 0.05 level.

**Table 4:** Estimated differences of single exposure in live birth at quantiles of ln-PFAS concentrations (ng/mL) measured in follicular fluid by generalized linear regression model (*n* = 208).

PFAS	OR (95%CI)				P for trend	Adjusted P^a^
	Q1	Q2	Q3	Q4		
CLP62	Reference	1.53 (0.67, 3.56)	1.34 (0.58, 3.11)	1.26 (0.54, 2.99)	0.67	0.87
CLP82	Reference	1.48 (0.64, 3.44)	1.38 (0.59, 3.29)	1.47 (0.64, 3.41)	0.43	0.65
HFPODA	Reference	1.18 (0.53, 2.67)	1.02 (0.44, 2.36)	1.08 (0.48, 2.44)	0.93	0.96
**PFBA**	Reference	0.67 (0.29, 1.51)	0.47 (0.20, 1.07)	**0.35 (0.14, 0.82)**	**0.01**	**0.04**
PFBS	Reference	1.55 (0.67, 3.63)	1.11 (0.47, 2.61)	0.72 (0.30, 1.71)	0.31	0.50
PFDA	Reference	1.67 (0.73, 3.88)	1.26 (0.54, 2.96)	1.35 (0.56, 3.27)	0.69	0.89
PFHpA	Reference	0.84 (0.36, 1.90)	1.00 (0.44, 2.27)	0.52 (0.22, 1.18)	0.19	0.43
PFHpS	Reference	3.56 (1.48, 8.93)	1.07 (0.45, 2.52)	1.59 (0.66, 3.88)	0.87	0.98
PFHxA	Reference	0.73 (0.32, 1.64)	0.46 (0.19, 1.12)	0.94 (0.41, 2.15)	0.69	0.89
nPFHxS	Reference	1.81 (0.78, 4.28)	1.52 (0.65, 3.58)	1.52 (0.64, 3.64)	0.43	0.65
PFNA	Reference	0.97 (0.42, 2.23)	1.25 (0.55, 2.85)	1.06 (0.46, 2.49)	0.75	0.96
PFOA	Reference	1.39 (0.60, 3.22)	1.84 (0.81, 4.29)	1.40 (0.58, 3.39)	0.34	0.51
nPFOS	Reference	1.48 (0.64, 3.47)	1.18 (0.51, 2.72)	0.92 (0.39, 2.20)	0.72	0.92
PFOS1m	Reference	2.76 (1.17, 6.71)	1.87 (0.81, 4.43)	1.36 (0.55, 3.42)	0.73	0.94
Mpfos345	Reference	1.72 (0.75, 4.01)	1.12 (0.48, 2.66)	1.25 (0.52, 3.02)	0.91	0.97
PFOS6m	Reference	2.16 (0.93, 5.16)	1.56 (0.67, 3.67)	1.05 (0.42, 2.57)	0.86	0.94
PFPeS	Reference	0.92 (0.39, 2.16)	1.84 (0.79, 4.34)	1.38 (0.59, 3.24)	0.23	0.49
PFUnDA	Reference	1.33 (0.56, 3.17)	1.10 (0.48, 2.54)	1.23 (0.52, 2.96)	0.76	0.95

PFAS, per- and poly-fluoroalkyl substances; OR, odds ratio; CI, confidence interval; BMI, body mass index. Models adjusted for women’s age at oocyte retrieval, paternal age, infertility duration, BMI, infertility diagnosis, parity, stimulation protocol.

a Adjusted with multiple hypothesis testing correction.

### 3.4 Associations between mixed PFASs and blastocyst quality and pregnancy outcomes

**Figure 1:**
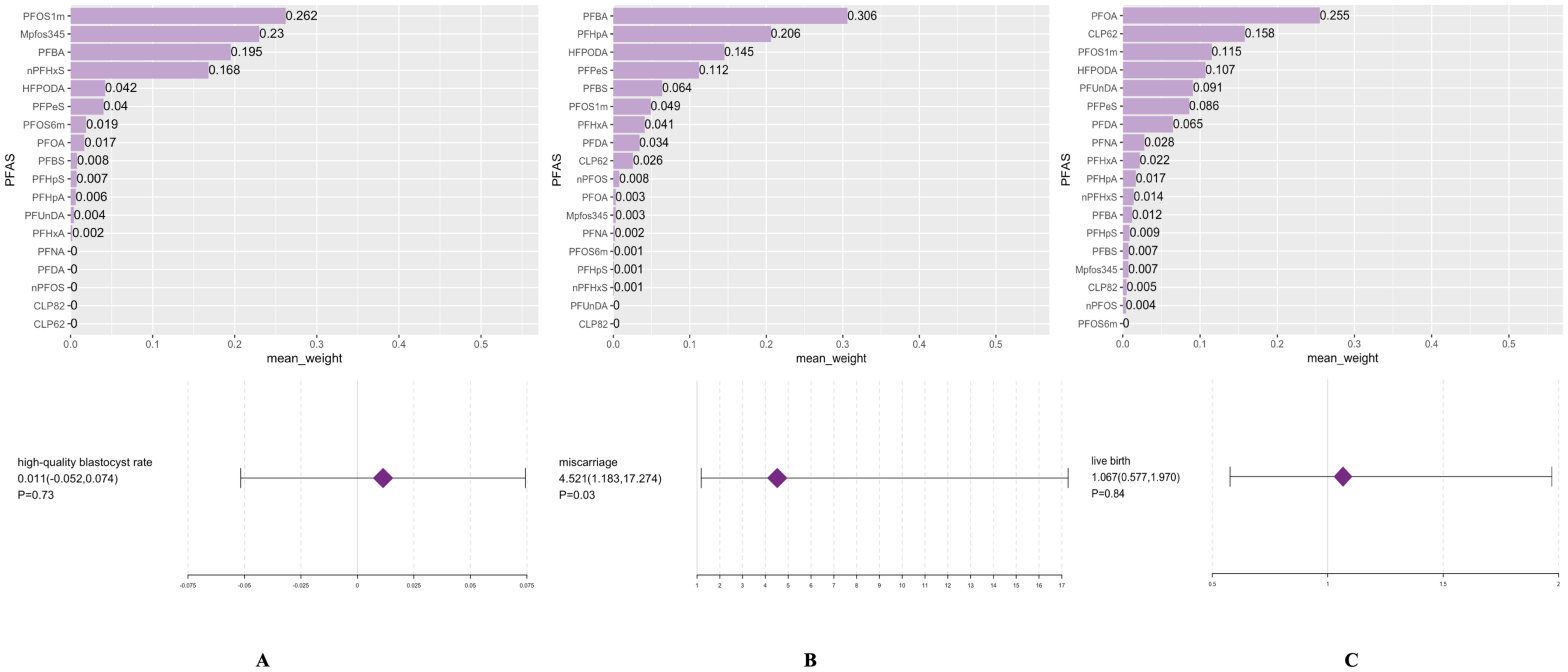
Mixed effect between PFAS exposure and embryo or pregnancy outcomes by weight quantile sum regression model. PFAS chemicals were sorted by weight in each weight quantile sum regression model. Panels **(A–C)** were used to estimate both the contribution of each PFAS congener and overall effect from mixed exposure on high-quality blastocyst rate, miscarriage and live birth, respectively.

**Figure 2:**
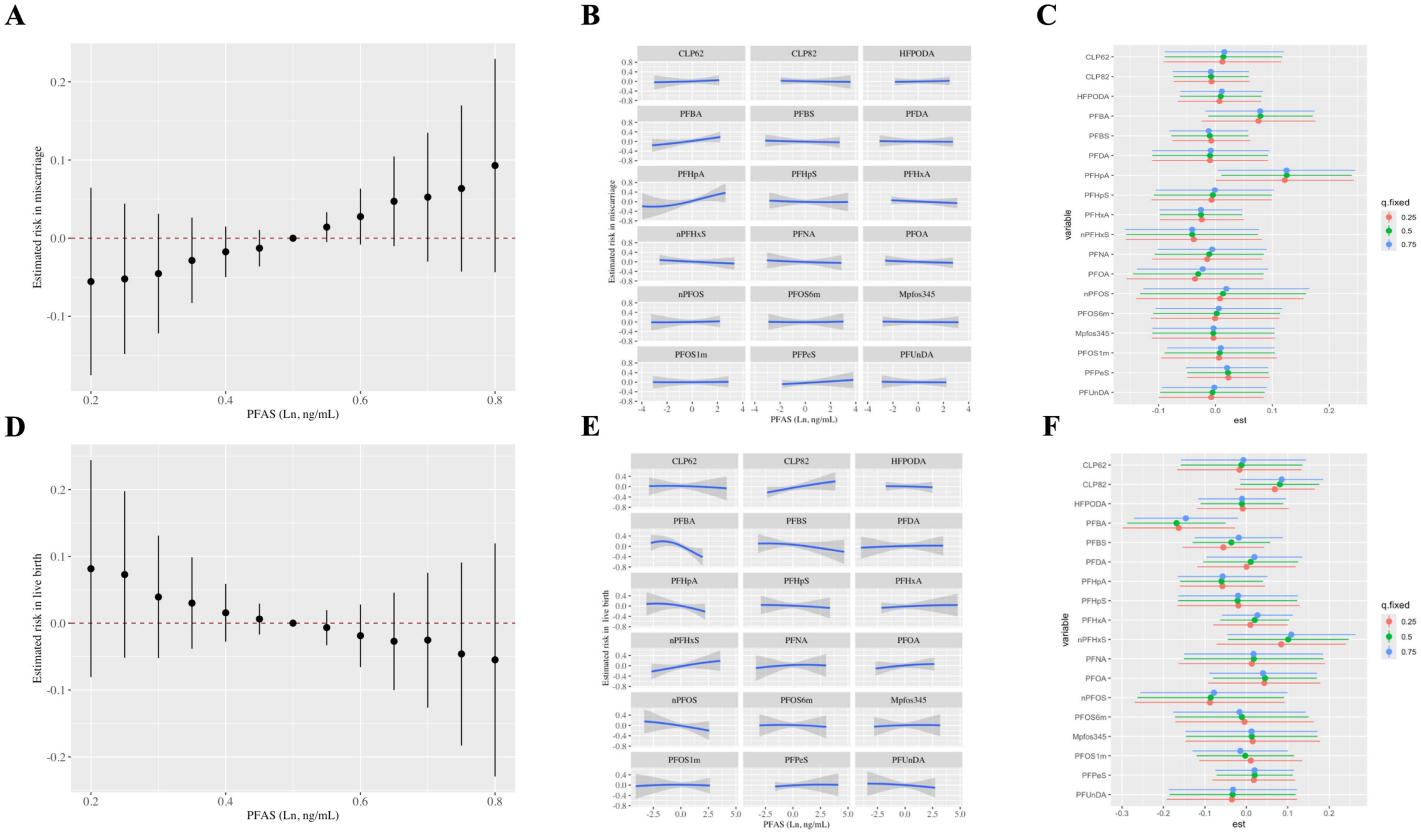
Mixed exposure between PFAS exposure and pregnancy outcomes by BKMR (iter = 10,000). The mixed effect curves of PFAS mixture on miscarriage **(A)** and live birth **(D)** when comparing all PFAS chemicals at particular percentiles were compared to all PFAS chemicals at their median. Univariate exposure-response curves (95% CI) for each PFAS chemical on miscarriage **(B)** and live birth **(E)**. Single exposure effect curves (95 %CI) for each PFAS chemical to miscarriage **(C)** and live birth **(F)** when other PFAS chemicals were fixed at a specific exposure percentile (25th, 50th, or 75th, respectively). Models adjusted for women’s age at oocyte retrieval, paternal age, infertility duration, BMI, infertility diagnosis, parity, stimulation protocol. PFAS, per- and poly-fluoroalkyl substances; BKMR, bayesian kernel machine regression; CI, confidence interval; BMI, body mass index.

To further verify the role of PFAS congeners in mixture, WQS and BKMR models were conducted for subsequent analysis. WQS models were presented in Figure 1 which showed a significant association between PFAS mixtures and an increased risk of miscarriage (OR: 4.52 (95%CI: 1.18, 17.28), p = 0.03) while no impact on high-quality blastocysts rate was observed (*β*: 0.01 (95%CI: −0.05, 0.07), *p* = 0.73). Weight contributions represented the relative importance of each PFAS chemical on the outcomes in the overall mixed models and were ranked from high to low. The highest weighted PFAS chemical for miscarriage was PFBA (30.6%), followed by PFHpA (20.6%) (Figure 1). Here, it is worth mentioning that no associations on live birth were exhibited. This lack of association may be attributable to the leading role of PFOA which showed the highest concentrations (Supplementary Table S2) and strong correlations with several other congeners (Supplementary Figure S3). These reasons may attenuate the significant effect of PFBA. In BKMR models, the overall effect on miscarriage and live birth was not significant. However, these models still revealed an increasing trend of miscarriage risk but decreasing trend of live birth rate with PFAS mixture concentrations (Figure 2). According to univariate dose–response curves, PFBA and PFHpA had a significant dose–response effect on miscarriage so did PFBA on live birth. When fixed at 25th, 50th or 75th percentiles, PFHpA was significantly correlated with increased risk of miscarriage and PFBA was strongly associated with decreased live birth rate. Analysis of posterior inclusion probability showed PFHpA, PFBA had the highest probability for miscarriage (PIP = 0.41, 0.18, respectively), whereas PFBA, PFHpA had the highest probability for live birth (PIP = 0.33, 0.31, respectively) (Supplementary Table S4). These results were consistent with results derived from generalized linear regression models.

## 4 Discussion

Our study detected 23 PFAS congeners from follicular fluid samples and found that PFAS exposure was associated with increased miscarriage risk and decreased live birth rate whereas having no impact on high-quality blastocysts rate. Among them, PFBA showed predominant effect on increasing miscarriage risk and decreasing live birth rate; PFHpA also exhibited a strong correlation with miscarriage.

The association between PFAS exposure and embryo quality or pregnancy outcomes has been debated in several studies with conclusions varying across research. Nevertheless, among these studies, most of them used serum samples for analysis. Although some studies identified high correlations between plasma and follicular fluid (7, 26), we considered follicular fluid better represented oocyte microenvironment which may reflect the biological effect of PFAS on quality of embryos more accurately.

Among studies using follicular fluid samples, some were consistent with our results (7, 16). For insistence, Kim et al. (16) assessed 97 matched samples from Australian women and reported no effect of PFAS on embryo quality. Hong et al. (7) originally aimed to compare serum and follicular fluid samples of 162 women undergoing IVF and determine the blood-follicle transfer efficiencies of PFAS. They additionally found no associations between PFAS exposure and selected IVF outcomes (including oocyte maturation rate, fertilization rate, high-quality cleavage embryo rate, biochemical pregnancy, clinical pregnancy and preclinical spontaneous abortion) but did not provide follow-up data until live birth. Notably, the study population in Hong et al.’s (7) study was geographically similar with ours which offered further support for our results. However, another study conducted by Zeng et al. (19) was contrary to ours. They enrolled 729 women in Guangxi province of China and pointed out that higher perfluoroalkyl acids concentrations in follicular fluid related to poorer embryo quality in IVF. The inconsistencies observed in Zeng et al.’s study may be attributable to demographic and geographical factors as nearly half of their study population were ethnic minorities of China whose distinct dietary habits and lifestyles may modify exposure outcomes or the population’s susceptibility might differ. Also, although the sample size of this study was currently the largest which focused on embryo quality, they did not provide data on either blastocyst quality and IVF pregnancy outcomes.

Our study was a prospective cohort study of fresh embryo transfer cycles focused on both embryo quality and pregnancy outcomes meanwhile using follicular fluid samples. In this population-based study, PFBA and PFHpA - two kinds of short-chain PFAS chemicals - were first reported for their miscarriage risk. Here, long- (≥8) or short – (<8) chain PFAS was classified by the carbon chain length. The additional carbon-fluorine bonds had lipophilicity and high affinity for protein binding which promoted the bioaccumulation of long-chain PFAS and caused long-term lipid metabolic dysfunction as well as hypothalamic–pituitary-gonadal axis disruption (35). In addition to these impacts, short-chain PFAS have been proved easier to cross kinds of biological barriers (26, 36, 37). Previous studies have linked short-chain PFAS to abnormal fetal sex hormone levels (38) and found specific accumulation in the decidua layer of the placenta (39). Also, with the short half-lives, repeated exposure and chronic injury may be easier for short-chain PFAS (40). Previously, Das et al. (36) conducted concentration gradient studies in murine models and suggested incidence of full-litter loss was significantly greater in the highest PFBA concentration group with no significance in number of implantation. PFHpA has been reported strongly correlated with fetal growth restriction (41–43) and abnormal fetal andogen indexs (38). It is also worth mentioning that only high-quality blastocysts of fresh cycles were transferred and these blastocysts had preferable viability. Therefore, although miscarriage mechanisms of PFBA and PFHpA have not been explored yet, maternal factors, placenta dysfunction may be vital reasons contributed to PFAS-associated miscarriage.

Endometrial dysfunction is usually the primary cause among maternal factors. As estrogen-like chemicals, PFASs have been identified with the function of altering some receptor activity (44, 45). Dixon et al. (45) explored PFASs modulated osmotic signaling pathway and exerted carcinogenic changes in rat uterus. Di Nisio et al. (44) found PFAS exposure led to a dysregulation of genetic cascade for endometrial receptivity and altered progesterone activity on endometrial cells. In addition, a few studies have demonstrated adverse effect of PFAS maternal exposure on both maternal and neonatal thyroid function which also caused abnormal pregnancy (46).

Placenta is also indispensable for maintaining normal pregnancy. However, PFAS exposure could change normal physiological structure of placenta (47, 48) as well as trophoblast function (49). Some studies have found that PFAS exposure increased the tortuosity of both spiral and basal arteries thereby affecting blood flow of placenta which further led to an increased fetal-plancental weight ratio, insufficient nutrition and growth restriction (47, 50). The expression of some markers such as vascular endothelial growth factor (51), the ratio of soluble fms like tyrosine kinase-1/placental growth factor-1 (52) were also found to be abnormally upregulated. Besides, PFAS impaired trophoblast functions such as migration, invasion and lipid homeostasis by reducing chemokine levels, downregulating inflammatory pathways, activating the peroxisome proliferator-activated receptors pathway and Notch signaling (49, 50, 53). While the specific molecular targets remain unclear and warrant further inverstigation, these findings highlight the need for public awareness on endometrial and placental health under PFAS exposure.

Of note, although a few population-based studies concluded similarly, they reported different PFAS congeners as risk factors in reproductive health. The discrepancies may lie in the difference of exposure modes and body fluid concentrations of participants from various regions. Thus, chemicals without significant results do not mean totally irrelevant with observed outcomes. Animal and cell experiment researches on minor PFAS should be conducted to further explore the toxicity. Also, given the potential reproductive toxicity of PFAS to ART population, future efforts should focus on expanding sample sizes such as conducting multi-center studies to set specific intervention thresholds. For highly-exposed population, regular monitoring for internal PFAS concentrations should be implemented. Measures such as strengthening the management of soil (54) and water (55), occupational protection (56) should be taken to reduce PFAS exposure.

The major strength of our study was that it analyzed PFAS concentrations from follicular fluid samples and focused on both embryo quality and pregnancy outcomes which are the most important concern for couples with infertility. Besides, this study firstly reported effect of PFAS exposure on blastocysts among population-based studies. The quality of blastocysts should be attached great importance as blastocyst embryo transfer is a trend in clinical practice and has been proved to be effective for live birth and preventing pregnancy loss (27). Our study also had some limitations. First of all, follicular fluid was aspirated only from the dominant follicle. However, pooled follicular fluid samples are much likely to be contaminated. A previous study found strong correlations among follicles within a woman (57). Therefore, we considered PFAS concentrations of dominant follicle to some extent could reflect the average PFAS concentrations in follicles. Second, this study was a single-center study and included ART population from northern China so women with risk factors of infertility in general population (e.g., women with advanced age or recurrent spontaneous abortion) and people living in similar geographical characteristics could be attached importance on exposure detection and individual protection while the generalizability to the general population was limited. Multicenter studies with general fertile women, diverse populations and larger sample size were needed to reduce selection bias and further validate and extend our findings. Third, we only collected the data on the number of high-quality blastocyst rate currently and did not have information on blastocyst grade (e.g., distribution and developmental/morphological parameters) thus whether PFAS exposure affects embryo development rate remains unknown. Studies are needed to further discover the influence of PFAS exposure on embryo development. Fourth, some else factors may be potential confounders such as routes of PFAS exposure (58) and lifestyle factors (e.g., smoking and alcohol consumption) (59). However, self-report data from each participant of our study showed none was smoking or drinking alcohol and other data was not collected in our study. Co-exposure to other environmental chemicals may also play a role in modifying the effect of PFAS on outcome measurements (60). Therefore, these factors should be considered in future studies and experiment researches are especially needed to explore the direct impact of PFAS exposure (60). At last, as the small sample size of pregnancy complications and adverse neonatal outcomes, it was difficult to identify the associations with PFAS exposure. Thus, we reported these cases in Supplementary Table S3 to provide evidence for further studies.

All in all, our study suggested that for women that underwent assisted reproductive fresh cycles, exposure to PFAS had a potential higher miscarriage risk and lower possibilities of live birth rather than jeopardizing embryo quality. PFBA and PFHpA were the most harmful PFAS congeners for miscarriage, PFBA was also the predominant harmful PFAS congener for live birth. The underlying mechanisms for PFAS-associated miscarriage still need further exploration.

## Data Availability

Requests to access the data analyzed in this study should be directed to Wei Zhou, zzsmile12@163.com.
