# Supplementary material for: Exposure to per- and poly-fluoroalkyl substances and associations with embryo quality and adverse pregnancy outcomes: a prospective cohort study
Source: Front Public Health. 2026 Mar 6;14:1783940. doi: 10.3389/fpubh.2026.1783940 (PMC13004126; doi:10.3389/fpubh.2026.1783940)
Supplement: Supplementary file 1 [file Table_1.DOCX]

**Supplementary Materials**

**Exposure to per- and poly-fluoroalkyl substances and associations with embryo quality and adverse pregnancy outcomes: a prospective cohort study**

**Authors:** Jiahui Wang^a*^, Zhe Li^b*^, Kuona Hu^c*^, Jingmei Hu^c^, Ting Jiang^c^, Jia Liao^c^, Qian Zhang^c^, Lijing Sun^c^, Linlin Cui^c^, Rong Chen^d#^, Tianxiang Ni^c#^, Wei Zhou^c#^

^a^ Department of Gynecology and Obstetrics, Peking Union Medical College Hospital, Chinese Academy of Medical Sciences & Peking Union Medical College, National Clinical Research Center for Obstetric & Gynecologic Diseases, Beijing, China; State Key Laboratory of Reproductive Medicine and Offspring Health, Center for Reproductive Medicine, Institute of Women, Children and Reproductive Health, Shandong University, 250012, China; National Research Center for Assisted Reproductive Technology and Reproductive Genetics, Shandong University, Jinan, Shandong, 250012, China; Key Laboratory of Reproductive Endocrinology (Shandong University), Ministry of Education, Jinan, Shandong, 250012, China; Shandong Technology Innovation Center for Reproductive Health, Jinan, Shandong, 250012, China; Shandong Provincial Clinical Research Center for Reproductive Health, Jinan, Shandong, 250012, China; Shandong Key Laboratory of Reproductive Medicine, Shandong Provincial Hospital Affiliated to Shandong First Medical University, Jinan, Shandong, 250012, China; Research Unit of Gametogenesis and Health of ART-Offspring, Chinese Academy of Medical Sciences (No. 2021RU001), Jinan, Shandong, 250012, China

^b^ Department of Urology, Shandong Provincial Hospital，Shandong University; Department of Ultrasound, Qilu Hospital of Shandong University

^c^ State Key Laboratory of Reproductive Medicine and Offspring Health, Center for Reproductive Medicine, Institute of Women, Children and Reproductive Health, Shandong University, 250012, China; National Research Center for Assisted Reproductive Technology and Reproductive Genetics, Shandong University, Jinan, Shandong, 250012, China; Key Laboratory of Reproductive Endocrinology (Shandong University), Ministry of Education, Jinan, Shandong, 250012, China; Shandong Technology Innovation Center for Reproductive Health, Jinan, Shandong, 250012, China; Shandong Provincial Clinical Research Center for Reproductive Health, Jinan, Shandong, 250012, China; Shandong Key Laboratory of Reproductive Medicine, Shandong Provincial Hospital Affiliated to Shandong First Medical University, Jinan, Shandong, 250012, China; Research Unit of Gametogenesis and Health of ART-Offspring, Chinese Academy of Medical Sciences (No. 2021RU001), Jinan, Shandong, 250012, China

^d^ Department of Gynecology and Obstetrics, Peking Union Medical College Hospital, Chinese Academy of Medical Sciences & Peking Union Medical College, National Clinical Research Center for Obstetric & Gynecologic Diseases, Beijing, China

* These three authors contributed equally to this work.

#Corresponding authors：

Author Name: Rong Chen

Address: Department of Obstetrics and Gynecology, Peking Union Medical College Hospital, Shuaifuyuan No.1, Dongcheng District, Beijing,100730, China

Phone: 0086-010-69151188

Email: chenrongpumch@163.com

ORCID ID: 0000-0001-8751-9590

Author Name: Tianxiang Ni

Address: Center for Reproductive Medicine, Cheeloo College of Medicine, Shandong University, 157 Jingliu Road, Jinan, 250001, Shandong, China

Phone: 0086-0031-85651190

Email: tianxiangni907@163.com

ORCID ID: 0000-0001-6184-2832

Author Name: Wei Zhou

Address: Center for Reproductive Medicine, Cheeloo College of Medicine, Shandong University, 157 Jingliu Road, Jinan, 250001, Shandong, China

Phone: 0086-18906402870

Email: zzsmile12@163.com

ORCID ID: 0009-0007-7443-8100

**Tables and Figures legend**

Table S1. Abbreviation of PFAS measurements.

Table S2. Limits of detection (LOD), detection frequency and concentrations (ng/mL) in follicular fluid for each PFAS chemical.

Table S3. Pregnancy complications and neonatal outcomes of study population (n=86).

Table S4. Estimated posterior inclusion probability for PFAS in relation to high-quality blastocyst rate, miscarriage and live birth in BKMR.

Figure S1. Flowchart of participants selection.

Figure S2. Direct acyclic graph (DAG) for the association between PFAS exposure and embryos quality and pregnancy outcomes.

Figure S3. Pearson correlation matrix for ln-PFAS concentrations in follicular fluid included in this study.

**Table S1. Abbreviation of PFAS measurements.**

| Abbreviation | Full name |
| --- | --- |
| CLP62 | 6:2 chlorinated polyfluorinated ether sulfonic acid |
| CLP82 | 8:2 chlorinated polyfluorinated ether sulfonic acid |
| HFPODA | Hexafluoropropylene oxide dimer acid |
| PFBA | Perfluoro-n-butanoic acid |
| PFBS | Potassium perfluoro-1-butanesulfonate |
| PFDA | Perfluoro-n-decanoic acid |
| PFDoA | Perfluorododecanoic acid |
| PFHpA | Perfluoro-n-heptanoic acid |
| PFHpS | Sodium perfluoro-1-heptanesulfonate |
| PFHxA | Perfluoro-n-hexanoic acid |
| nPFHxS | Linear perfluorohexanesulfonate |
| BrPFHxS | Branched perfluorohexanesulfonate |
| PFNA | Perfluoro-n-nonanoic acid |
| PFOA | perfluoro-n-octanoic acid |
| nPFOS | Linear sodium perfluoro-1-octanesulfonate |
| PFOS1m | Potassium perfluoro-1-methylheptanesulfonate |
| Mpfos345 | Potassium perfluoro-3-methylheptanesulfonate + Potassium perfluoro-4-methylheptanesulfonate + Potassium perfluoro-5-methylheptanesulfonate |
| PFOS6m | Potassium perfluoro-6-methylheptanesulfonate |
| PFPeS | Sodium perfluoro-1-pentanesulfonate |
| PFPeA | Perfluoro-n-pentanoic acid |
| PFUnDA | Perfluoro-n-undecanoic acid |

**Table S2. Limits of detection (LOD), detection frequency and concentrations (ng/mL) in follicular fluid for each PFAS chemical.**

| PFAS | LOD | LOD / √2 | LOD ratio (%) | Median (IQR) | Q1 | | Q2 | | Q3 | | Q4 | |
| --- | --- | --- | --- | --- | --- | --- | --- | --- | --- | --- | --- | --- |
|  | (ng/mL) |  |  | (ng/mL) | Range | N | Range | N | Range | N | Range | N |
| CLP62 | 0.0008 | 0.0006 | 100% (246/246) | 1.136(0.695,2.095) | [0.009,0.695) | 62 | [0.695,1.136) | 61 | [1.136,2.095) | 61 | [2.095,36.862] | 62 |
| CLP82 | 0.00039 | 0.0003 | 100% (246/246) | 0.012(0.008,0.022) | [0.001,0.008) | 62 | [0.008,0.012) | 61 | [0.012,0.022) | 61 | [0.022,0.645] | 62 |
| HFPODA | 0.001 | 0.0007 | 87.40% (215/246) | 0.019(0.006,0.045) | [0.001,0.006) | 62 | [0.006,0.019) | 61 | [0.019,0.045) | 61 | [0.045,1.033] | 62 |
| PFBA | 0.0025 | 0.0018 | 93.09% (229/246) | 0.071(0.038,0.108) | [0.002,0.038) | 62 | [0.038,0.071) | 61 | [0.071,0.108) | 61 | [0.108,0.646] | 61 |
| PFBS | 0.005 | 0.0035 | 99.59% (245/246) | 0.040(0.029,0.063) | [0.004,0.029) | 62 | [0.029,0.040) | 61 | [0.040,0.063) | 61 | [0.063,2.084] | 62 |
| PFDA | 0.0061 | 0.0043 | 100% (246/246) | 0.329(0.208,0.620) | [0.009,0.208) | 62 | [0.208,0.329) | 61 | [0.329,0.620) | 61 | [0.620,7.101] | 62 |
| PFDoA^*^ | 0.015 | 0.0106 | 82.11% (202/246) | - | - | - | - | - | - | - | - | - |
| PFHpA | 0.0021 | 0.0015 | 94.72% (233/246) | 0.165(0.099,0.287) | [0.001,0.099) | 62 | [0.099,0.165) | 61 | [0.165,0.287) | 61 | [0.287,3.687] | 62 |
| PFHpS | 0.0007 | 0.0005 | 99.59% (245/246) | 0.049(0.030,0.082) | [0.000,0.030) | 62 | [0.030,0.049) | 61 | [0.049,0.082) | 61 | [0.082,0.634] | 62 |
| PFHxA | 0.0017 | 0.0012 | 96.34% (237/246) | 0.008(0.005,0.012) | [0.001,0.005) | 62 | [0.005,0.008) | 61 | [0.008,0.012) | 61 | [0.012,0.658] | 62 |
| nPFHxS | 0.0035 | 0.0025 | 100%(246/246) | 0.282(0.164,0.570) | [0.025,0.164) | 62 | [0.164,0.282) | 61 | [0.282,0.570) | 61 | [0.570,9.549] | 62 |
| BrPFHxS^*^ | 0.00945 | 0.0067 | 78.05% (192/246) | - | - | - | - | - | - | - | - | - |
| PFNA | 0.0052 | 0.0037 | 100% (246/246) | 0.554(0.355,0.898) | [0.013,0.355) | 62 | [0.355,0.554) | 61 | [0.554,0.898) | 61 | [0.898,6.000] | 62 |
| PFOA | 0.00454 | 0.0032 | 100% (246/246) | 4.891(2.902,10.356) | [0.017,2.902) | 62 | [2.902,4.891) | 61 | [4.891,10.356) | 61 | [10.356,56.717] | 62 |
| nPFOS | 0.0035 | 0.0025 | 100% (246/246) | 1.928(1.270,3.397) | [0.161,1.270) | 62 | [1.270,1.928) | 61 | [1.928,3.397) | 61 | [3.397,13.973] | 62 |
| PFOS1m | 0.0012 | 0.0008 | 99.19% (244/246) | 0.060(0.035,0.098) | [0.001,0.035) | 62 | [0.035,0.060) | 61 | [0.060,0.098) | 61 | [0.098,0.786] | 62 |
| Mpfos345 | 0.0086 | 0.0061 | 100%(246/246) | 0.319(0.189,0.515) | [0.0021,0.189) | 62 | [0.189,0.319) | 61 | [0.319,0.515) | 61 | [0.515,3.921] | 62 |
| PFOS6m | 0.005 | 0.0035 | 100% (246/246) | 0.179(0.101,0.278) | [0.015,0.101) | 62 | [0.101,0.179) | 61 | [0.179,0.278) | 61 | [0.278,1.981] | 62 |
| PFPeS | 0.001 | 0.0007 | 92.28% (227/246) | 0.004(0.002,0.006) | [0.001,0.002) | 62 | [0.002,0.004) | 61 | [0.004,0.006) | 61 | [0.006,0.347] | 62 |
| PFPeA* | 0.0017 | 0.0012 | 67.89% (167/246) | - | - | - | - | - | - | - | - | - |
| PFUnDA | 0.01 | 0.0071 | 100% (246/246) | 0.272(0.173,0.441) | [0.017,0.173) | 62 | [0.173,0.272) | 57 | [0.272,0.441) | 65 | [0.441,2.738] | 62 |

*Note*: ^*^ PFAS congeners whose detection rate below 85% were excluded for further analysis. PFAS, per- and poly-fluoroalkyl substances; IQR, interquartile range; LOD, limit of detection.

**Table S3.** **Pregnancy complications and neonatal outcomes of study population (n=86).**

|  | Values |
| --- | --- |
| **Pregnancy complications** |  |
| Gestational diabetes ^a^ | 12 (14.0) |
| Pregnancy-induced hypertension ^a^ | 6 (7.0) |
| **Neonatal outcomes** |  |
| Gestational weeks at birth ^b^ | 38.7 (37.2, 39.7) |
| Preterm birth ^a^ | 17 (19.8) |
| Infant sex ^a^ |  |
| Male | 58 (59.2) |
| Female | 40 (40.8) |
| Birth weight, grams ^b^ | 3175.0 (2762.5, 3587.5) |
| Low birth weight ^a^ | 19 (19.4) |

*Note:* The above information was self-reported. Generally, gestational diabetes was diagnosed based on oral glucose tolerance test during 24-28 weeks’ gestation and at least one of the following thresholds should be met: fasting blood glucose ≥5.1 mmol/L, 1-hour blood glucose ≥10.0 mmol/L, or 2-hour blood glucose ≥8.5 mmol/L. Pregnancy-induced hypertension was defined as a systolic blood pressure of ≥ 140 mmHg and a diastolic blood pressure of ≥ 90 mmHg and first diagnosed after the 20 weeks’ gestation. Preterm birth was defined as a delivery before 37 weeks’ gestation. Low birth weight was defined as infant weight at birth below 2500 grams.

^a^: n (%).

^b^: median (interquartile range).

**Table S4. Estimated posterior inclusion probability for PFAS in relation to high-quality blastocyst rate, miscarriage and live birth in BKMR.**

| PFAS | Estimated PIP | | |
| --- | --- | --- | --- |
|  | High-quality blastocyst rate | Miscarriage | Live birth |
| CLP62 | 0.1920 | 0.0862 | 0.2252 |
| CLP82 | 0.1690 | 0.0568 | 0.1902 |
| HFPODA | 0.1998 | 0.1014 | 0.0970 |
| PFBA | 0.1932 | 0.1798 | 0.3274 |
| PFBS | 0.2912 | 0.1216 | 0.2778 |
| PFDA | 0.1988 | 0.1158 | 0.1634 |
| PFHpA | 0.3078 | 0.4140 | 0.3096 |
| PFHpS | 0.1922 | 0.1692 | 0.1406 |
| PFHxA | 0.1970 | 0.0782 | 0.1244 |
| nPFHxS | 0.1612 | 0.1248 | 0.1516 |
| PFNA | 0.1948 | 0.1114 | 0.1634 |
| PFOA | 0.2070 | 0.1622 | 0.1806 |
| nPFOS | 0.2556 | 0.1536 | 0.2502 |
| PFOS1m | 0.1824 | 0.0490 | 0.1762 |
| Mpfos345 | 0.1422 | 0.1150 | 0.1328 |
| PFOS6m | 0.1770 | 0.1538 | 0.2064 |
| PFPeS | 0.1578 | 0.0796 | 0.1360 |
| PFUnDA | 0.2006 | 0.1164 | 0.1864 |

*Note*: PFAS, per- and poly-fluoroalkyl substances; PIP, posterior inclusion probability; BMI, body mass index. Models adjusted for women’s age at oocyte retrieval, paternal age, infertility duration, BMI, infertility diagnosis, parity, stimulation protocol.

**Figure S1. Flowchart of participants selection.**


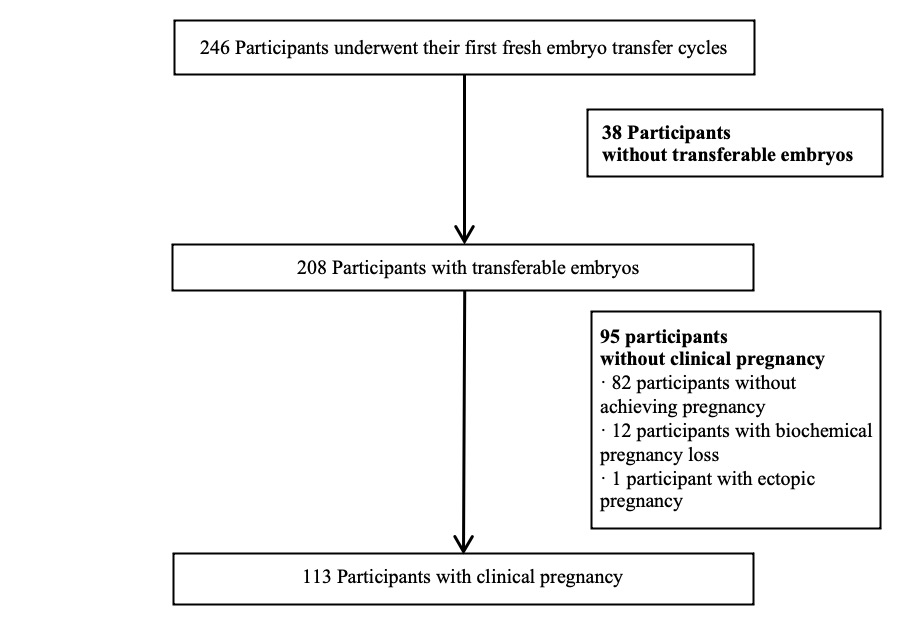


**Figure S2. Direct acyclic graph (DAG) for the association between PFAS exposure and embryos quality and pregnancy outcomes.**

**
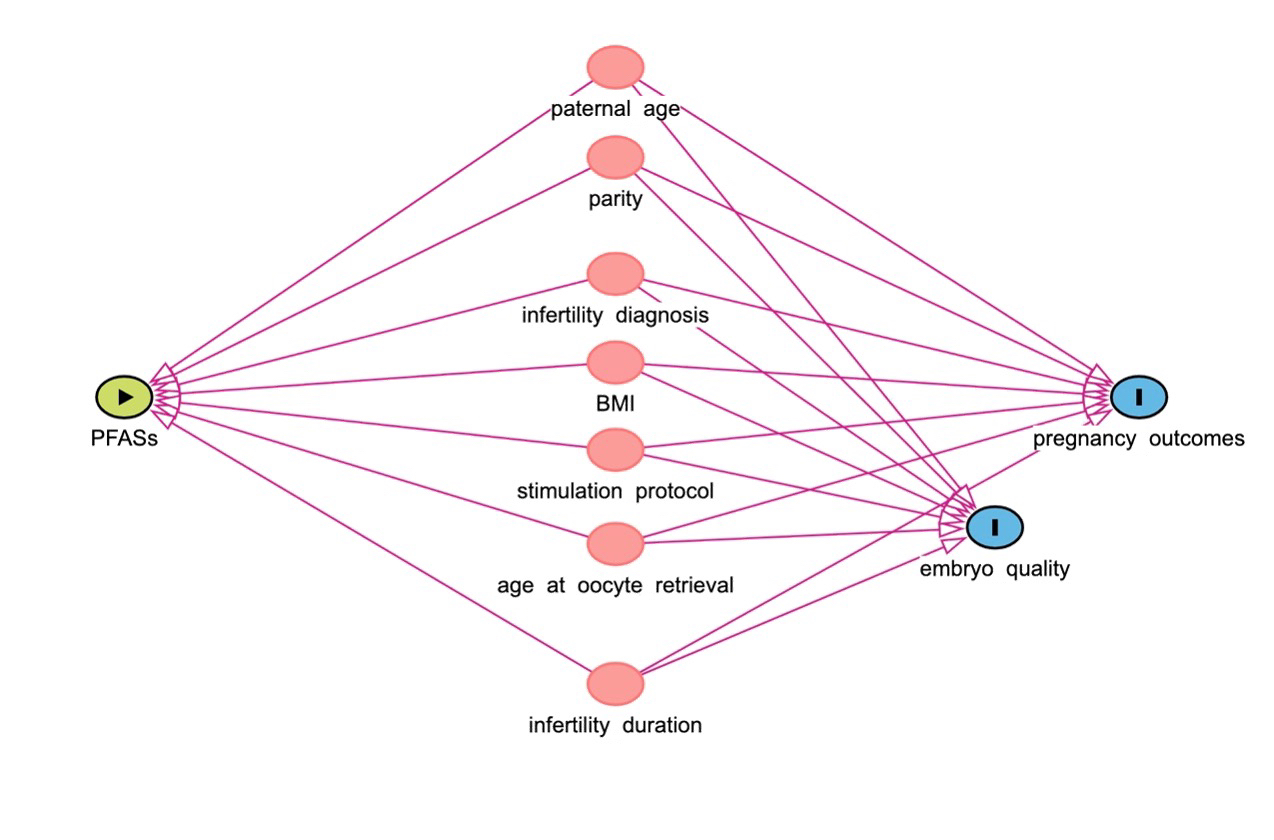
**

*Note*: PFAS, per- and poly-fluoroalkyl substances; BMI, body mass index. Green circle represents exposure, red circle represents adjusted confounders, blue circle represents outcomes.

**Figure S3. Pearson correlation matrix for ln-PFAS concentrations in follicular fluid included in this study.**
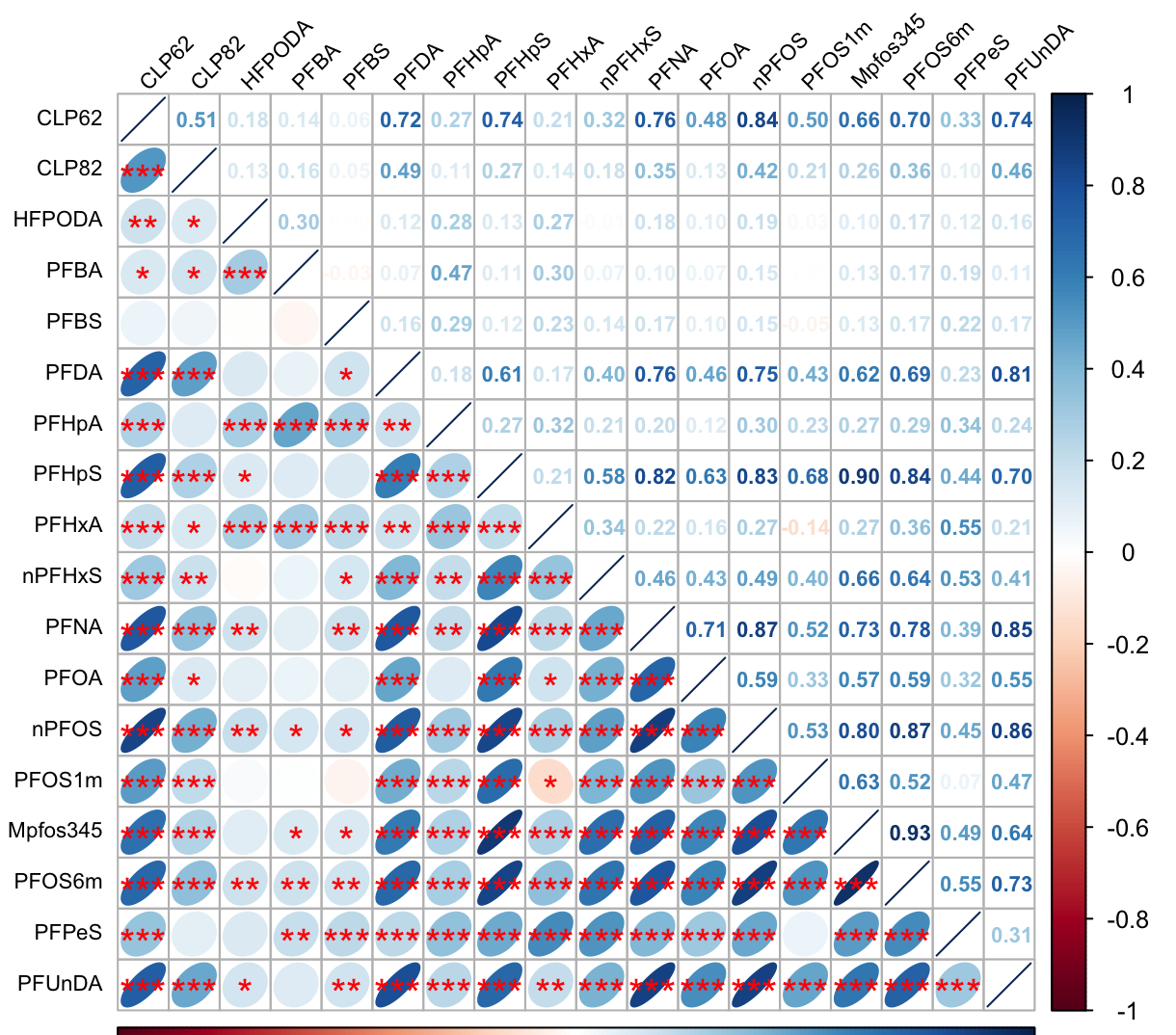


*Note*: p<0.05 was marked with an asterisk, p<0.01 was marked with 2 asterisks, p<0.001 was marked with 3 asterisks)
